# Swimming Baths for Children

**Published:** 1890-10

**Authors:** 


### SWIMMING BATHS FOR CHILDREN.

It is now generally agreed that young children should not be taught to swim, as they are so susceptible to fatigue and cold, and so generally impressionable. Up to two or three years, prolonged immersion in cold, or even warm water is not safe. Much, however, depends on the temperament of the child in question ; if placid, fearless, and healthy, he might occasionally be given a swimming lesson, but he should not remain in the water at first more than six or eight minutes, nor should he be given a lesson oftener than once a week, or at most twice. Should he show dread of the water, and cry or struggle much, he should not be taken to it a second time till the summer of next year ; the most serious results might otherwise be the consequence. Should he be cold after immersion, restless, or lose his appetite, that, too, would be an indication to be careful. A wise and thoughtful parent will learn as quickly as any doctor what the little fellow can or cannot safely bear. Custom and convenience generally fix upon the early morning as the best time for a cold bath, and yet, experience shows that this is not by any means the best part of the day ; and it is said that about the middle of the day, when breakfast has been thoroughly digested, while the system is still fresh, is the most reasonable part of the day. I do not, however, think that it is probable that a child of three and a half can be taught to swim, even in summer, without some risk and difficulty, though I cannot see any objection to the experiment being cautiously tried.

